# Estimated Dietary Polyphenol Intake and Major Food and Beverage Sources among Elderly Japanese

**DOI:** 10.3390/nu7125530

**Published:** 2015-12-09

**Authors:** Chie Taguchi, Yoichi Fukushima, Yoshimi Kishimoto, Norie Suzuki-Sugihara, Emi Saita, Yoshinari Takahashi, Kazuo Kondo

**Affiliations:** 1 Endowed Research Department “Food for Health”, Ochanomizu University, 2-1-1 Otsuka, Bunkyo-ku, Tokyo 112-8610, Japan; taguchi.chie@ocha.ac.jp (C.T.); saita.emi@ocha.ac.jp (E.S.); kondo.kazuo@ocha.ac.jp (K.K.); 2 Nestlé Japan Ltd., NYK Tennoz Blidg., 2-2-20 Higashi-Shinagawa, Shinagawa-ku, Tokyo 140-0002, Japan; yoichi.fukushima@jp.nestle.com; 3 Department of Nutrition and Food Science, Graduate School of Humanities and Sciences, Ochanomizu University, 2-1-1 Otsuka, Bunkyo-ku, Tokyo 112-8610, Japan; g1370512@edu.cc.ocha.ac.jp; 4 TES Holdings Co., Ltd., 6F Tokyo University Entrepreneurs Plaza, 7-3-1 Hongo, Bunkyo-ku, Tokyo 113-0033, Japan; y.takahashi@tes-h.co.jp; 5 Institute of Life Innovation Studies, Toyo University, 1-1-1 Izumino, Itakura-machi, Ora-gun, Gunma 374-0193, Japan

**Keywords:** polyphenol, consumption, beverage, food, elderly, coffee, green tea

## Abstract

Estimating polyphenol intake contributes to the understanding of polyphenols’ health benefits. However, information about human polyphenol intake is scarce, especially in the elderly. This study aimed to estimate the dietary intake and major sources of polyphenols and to determine whether there is any relationship between polyphenol intake and micronutrient intake in healthy elderly Japanese. First, 610 subjects (569 men, 41 women; aged 67.3 ± 6.1 years) completed food frequency questionnaires. We then calculated their total polyphenol intake using our polyphenol content database. Their average total polyphenol intake was 1492 ± 665 mg/day, the greatest part of which was provided by beverages (79.1%). The daily polyphenol intake differed largely among individuals (183–4854 mg/day), also attributable mostly to beverage consumption. Coffee (43.2%) and green tea (26.6%) were the major sources of total polyphenol; the top 20 food items accounted for >90%. The polyphenol intake did not strongly correlate with the intake of any micronutrient, suggesting that polyphenols may exert health benefits independently of nutritional intake. The polyphenol intake in this elderly population was slightly higher than previous data in Japanese adults, and beverages such as coffee and green tea contributed highly to the intake.

## 1. Introduction

Polyphenols are present in high amounts in most plant foods and beverages [1], and cannot be synthesized by humans [2]. In the 1990s, several epidemiological studies demonstrated that dietary polyphenol consumption is associated with a reduced risk of cardiovascular disease [3,4]. As the basic and clinical research progressed, multiple functions of polyphenols contributing to human health were identified [5,6]. To elucidate the contribution of polyphenols to human health, it is necessary to estimate individuals’ polyphenol intake in their daily diet. Comprehensive databases of the polyphenol content of food such as the United States Department of Agriculture (USDA) Database for the Flavonoid Content of Selected Foods [7] and the Phenol-Explorer databases [8,9] by the French National Institutes for Agricultural Research have been established and used for estimating the individuals’ polyphenol intake in European and American countries [10,11,12,13]. Through the estimation of dietary polyphenols, several cohort studies have shown an inverse association between polyphenol intake and the risk of chronic diseases such as cardiovascular diseases [14,15,16,17,18,19,20], cancers [21,22,23,24,25], and all-cause mortality [26]. In a recent publication from the PREDIMED trial, a 37% reduction of mortality was observed in a comparison of extreme quintiles of total polyphenol intake [27]. However, information about polyphenol intake in Japanese populations is still limited [28].

Japan has a unique food culture, and thus a database on the polyphenol contents of foods consumed in Japan is needed to estimate the daily polyphenol intake in Japanese. We measured total polyphenol contents by conducting a modified Folin-Ciocalteu colorimetric assay for 77 food and beverage items, and established a database for the polyphenol content. Using the database, we have assessed the daily polyphenol intake in the following Japanese populations: (1) randomly selected male and female subjects (10–59 years old, *n* = 8768) who recorded all non-alcoholic beverages consumed in a week [29]; (2) 109 middle-aged Japanese women who recorded all beverages and foods ingested in a week [30]; and (3) randomly selected male and female subjects (1–99 years old, *n* > 10,000 every year for 18 years) who recorded all beverages consumed in a week [31]. According to these studies, we documented that coffee and green tea are the major contributors to total polyphenol consumption [29,30,31]. However, there is very limited information about the dietary polyphenol intake among elderly Japanese.

The elderly population has been growing rapidly both in Japan and worldwide. Polyphenols have been recognized to be beneficial for elderly people, to prevent chronic diseases and maintain health. It was recently reported that dietary polyphenol might be associated with lower risks of substantial cognitive decline [32] and frailty [33] in older adults. A polyphenol-rich diet also has potential for increasing specific nutrients, because plant foods and beverages are also the main sources of vitamins, minerals or fiber in the diet. However, the relationship between dietary polyphenol and nutrients has not been elucidated.

This study was conducted to (1) estimate the polyphenol intake among elderly Japanese; (2) determine the contributions of specific foods to the total polyphenol intake; and (3) clarify the relationships between polyphenol and micronutrient intake.

## 2. Experimental Section

### 2.1. Study Population

We posted two questionnaires by mail to 986 Japanese listed as retired employees of Nestlé Japan Ltd., and from 685 replies we obtained 627 subjects who completed two questionnaires. We excluded 17 subjects whose energy intake was either too low (at less than one-half the energy requirement for the lowest physical activity category) or too high (at 1.5 times more than the energy requirement of the highest physical activity category), referring to the Dietary Reference Intakes for Japanese (2010) [34]. Thus, 610 subjects (569 men and 41 women) aged 52–89 (67.3 ± 6.1 years) were included in this analysis.

### 2.2. Estimation of Dietary Polyphenol

The subjects’ dietary habits during the previous month were assessed using two different food frequency questionnaires (FFQs). One was our originally developed FFQ for polyphenol intake from food and beverages that assesses the intake of 22 foods and 23 beverages, which are significant items rich in polyphenol according to our previous studies [29,30]. The other FFQ was a brief-type self-administered diet history questionnaire (BDHQ), which assesses the intake frequency of 58 food and beverage items [35,36]. Each subject’s consumption of each food item was obtained using the two FFQs. The intakes of energy and nutrients were estimated using the intakes of food items described in the BDHQ. The subjects filled out the two FFQs in September or October of 2012.

In this study, we used our original database of the polyphenol content of 67 items: 15 types of beverages and 52 types of foods, mostly described in previous reports [29,30]. The polyphenol contents of chocolate were revised as 390 mg/100 g and 1560 mg/100 g, respectively, for milk chocolate (5% cacao mass) and bitter chocolate (20% cacao mass) from the previous values (935.5 mg/100 g for 12% cacao mass) [30]. We calculated the polyphenol intake by matching the food consumption data from two FFQs with the polyphenol content in the food and beverages described above.

### 2.3. Statistical Analysis

All data are expressed as mean ± standard deviation (SD). Statistical analyses were performed with IBM SPSS software, version 20. Spearman’s correlation coefficients were calculated to assess the relationships between variables.

## 3. Results

### 3.1. Subjects’ Characteristics

The characteristics of the 610 subjects are shown in Table 1. As noted above, the mean age of the 569 men and 41 women was 67.3 ± 6.1 years (range 52–89 years). The average body mass index (BMI) was 23.1 ± 2.6 kg/m^2^ in the men and 22.3 ± 2.8 kg/m^2^ in the women. The estimated daily energy was 2185 ± 594 kcal/day in the men and 1670 ± 475 kcal/day in the women. The average dietary energy and nutrient intakes were almost the same as the average Japanese intakes referred to in a recent National Health and Nutrition Survey [37] and were of almost sufficient quantity to meet the Adequate Intake of Dietary Reference Intakes for Japanese (2010) [34].

**Table 1:** Characteristics of the population (569 men and 41 women) and their nutritional intake.

		Men and Women (*n* = 610)		Men (*n* = 569)		Women (*n* = 41)	
		Mean ± SD	Min–Max	Mean ± SD	Min–Max	Mean ± SD	Min–Max
Age	years	67.3 ± 6.1	(52–89)	67.3 ± 6.0	(55–89)	66.7 ± 7.2	(52–81)
Body weight	kg	64.3 ± 8.8	(35.0–95.0)	65.0 ± 8.4	(46.0–95.0)	54.0 ± 8.0	(35.0–69.0)
BMI	kg/m^2^	23.1 ± 2.7	(15.1–34.5)	23.1 ± 2.6	(16.6–34.5)	22.3 ± 2.8	(15.1–28.6)
Energy	kcal	2150 ± 600	(895–4035)	2185 ± 594	(1031–4035)	1670 ± 475	(895–2898)
Protein	% energy	15.5 ± 3.1	(8.9–28.1)	15.4 ± 3.1	(8.9–28.1)	16.9 ± 3.1	(10.7–24.3)
Fat	% energy	26.4 ± 5.1	(14.5–43.5)	26.2 ± 5.1	(14.5–43.5)	29.4 ± 4.7	(21.5–42.8)
Carbohydrate	% energy	58.1 ± 7.4	(33.3–75.9)	58.4 ± 7.3	(33.3–75.9)	53.7 ± 6.7	(38.2–65.3)

### 3.2. Polyphenol Intake

**Figure 1:**
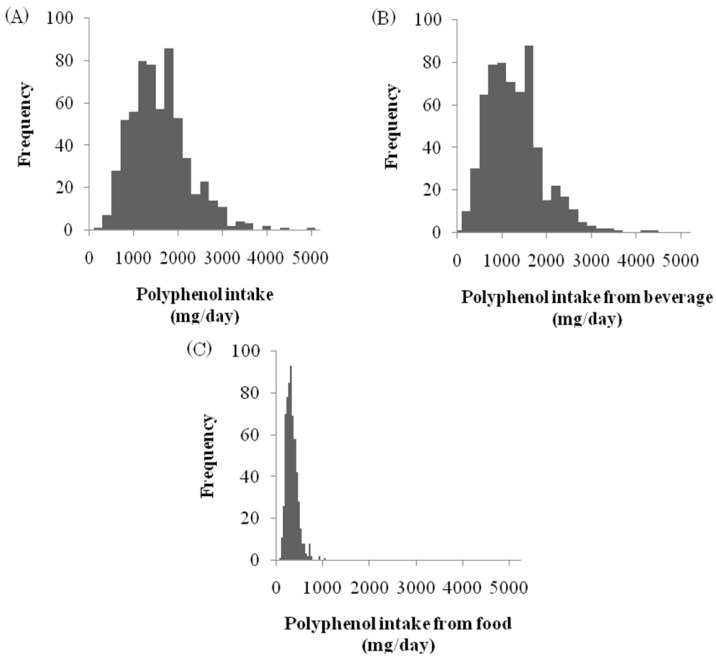
Histograms of total polyphenol intake (**A**); polyphenol intake from beverages (**B**); and polyphenol intake from foods (**C**).

The polyphenol intake is summarized in Figure 1 and Table 2. The subjects consumed 1492 ± 665 mg/day of polyphenols on average (range 183–4854 mg/day). The average polyphenol intake from beverages was 1180 ± 629 mg/day, accounting for 79.1% of the total polyphenol intake, and the average polyphenol intake from food was 312 ± 126 mg/day, accounting for 20.9%. The polyphenol intakes from non-alcoholic beverages and alcoholic beverages were 1104 ± 607 mg/day (74.0%) and 76 ± 166 mg/day (5.1%), respectively. Of the food groups, the portions of the polyphenol intake provided by vegetables was 6.8%; that by cereals, 3.2%; fruits, 3.0%; beans, 2.9%; and seasonings, 2.7%. For both the men and women, approx. 80% of their polyphenol intake was from beverages and approx. 20% was from food.

The proportions and ranking of the polyphenol intake in this elderly Japanese population are shown in Table 3. Only small differences between men and women in the contribution of various food items was apparent. Although green tea was the most consumed beverage at 345 ± 306 mL/day (approx. 2.3 cups) and coffee was the second largest one at 329 ± 263 mL/day (approx. 2.2 cups), coffee was the largest source of polyphenol intake from all food and beverages, accounting for 43.2%. Green tea, the second largest source, accounted for 26.6%. The two major beverages together accounted for approx. 70%, and beverages other than green tea and coffee accounted for at most 3%. The third largest source of polyphenols in the men was beer at 3.0% (in the women, beer ranked 30th at 0.3%), and the third largest source of polyphenols in the women was black tea at 4.6% (in the men, black tea ranked 10th at 1.4%).

**Table 2:** The subjects’ polyphenol intake from beverages and foods.

	Men and Women (*n* = 610)			Men (*n* = 569)			Women (*n* = 41)		
	Polyphenol Intake (mg/Day)		Polyphenol Intake (%)	Polyphenol Intake (mg/Day)		Polyphenol Intake (%)	Polyphenol Intake (mg/Day)		Polyphenol Intake (%)
	Mean ± SD	Min–Max		Mean ± SD	Min–Max		Mean ± SD	Min–Max	
Total	1492 ± 665	(183–4854)	100%	1503 ± 669	(183–4854)	100%	1326 ± 583	(410–2970)	100%
Beverages	1180 ± 629	(0–4380)	79.1%	1190 ± 634	(0–4380)	79.1%	1040 ± 545	(252–2497)	78.4%
Non alcoholic beverages	1104 ± 607	(0–4058)	74.0%	1110 ± 611	(0–4058)	73.8%	1025 ± 553	(118–2497)	77.3%
Alcoholic beverages	76 ± 166	(0–2307)	5.1%	80 ± 171	(0–2307)	5.3%	15 ± 32	(0–134)	1.2%
Foods	312 ± 126	(71–1013)	20.9%	314 ± 127	(71–1013)	20.9%	286 ± 101	(114–543)	21.6%
Vegetables	102 ± 63	(8–457)	6.8%	102 ± 63	(8–457)	6.8%	95 ± 58	(24–312)	7.2%
Cereals	47 ± 31	(0–190)	3.2%	49 ± 31	(0–190)	3.2%	30 ± 15	(6–79)	2.3%
Fruits	44 ± 35	(0–209)	3.0%	44 ± 35	(0–209)	2.9%	49 ± 33	(6–119)	3.7%
Pulses	44 ± 33	(0–212)	2.9%	44 ± 33	(0–212)	2.9%	45 ± 31	(5–146)	3.4%
Seasonings	40 ± 13	(12–104)	2.7%	41 ± 13	(12–104)	2.7%	31 ± 13	(12–72)	2.4%
Confectioneries	14 ± 27	(0–390)	0.9%	14 ± 27	(0–390)	0.9%	13 ± 15	(0–61)	1.0%
Nuts and seeds	14 ± 14	(0–116)	0.9%	14 ± 14	(0–116)	0.9%	17 ± 14	(0–53)	1.3%
Potatoes	4 ± 4	(0–29)	0.3%	4 ± 4	(0–29)	0.3%	4 ± 3	(0–10)	0.3%
Algae	2 ± 2	(0–15)	0.1%	2 ± 2	(0–15)	0.1%	2 ± 2	(0–5)	0.1%

**Table 3:** The proportion and ranking of polyphenol intake in the subjects.

		Type	Polyphenol Intake (mg/Day)		Polyphenol Intake (%)	Cumulative Polyphenol Intake (%)
			Mean ± SD	Min–Max		
**Men and women (*n* = 610)**						
1	Coffee	B	645 ± 517	(0–2613)	43.2%	43.2%
2	Green tea	B	397 ± 351	(0–1380)	26.6%	69.9%
3	Beer	B	43 ± 63	(0–513)	2.9%	72.7%
4	Red wine	B	33 ± 152	(0–2243)	2.2%	74.9%
5	Tomato vegetable juice	V	28 ± 44	(0–311)	1.9%	76.8%
6	Spinach/broccoli	V	26 ± 18	(0–107)	1.7%	78.5%
7	Soy sauce	S	23 ± 7	(8–51)	1.6%	80.1%
8	Black tea	B	23 ± 62	(0–432)	1.6%	81.6%
9	Buckwheat noodle	C	22 ± 26	(0–162)	1.5%	83.1%
10	*Natto*	P	21 ± 23	(0–142)	1.4%	84.5%
11	*Tofu*/fried *tofu*	P	20 ± 13	(0–77)	1.3%	85.9%
12	Oolong tea	B	19 ± 55	(0–468)	1.3%	87.2%
13	Onion/welsh onion	V	17 ± 12	(0–70)	1.1%	88.3%
14	Orange	F	16 ± 19	(0–121)	1.1%	89.4%
15	*Miso*	S	14 ± 10	(0–61)	1.0%	90.4%
16	Bread	C	14 ± 9	(0–46)	1.0%	91.3%
17	Chocolate	Co	14 ± 27	(0–390)	0.9%	92.2%
18	Barley tea	B	13 ± 23	(0–108)	0.9%	93.1%
19	Strawberry	F	11 ± 14	(0–111)	0.8%	93.9%
20	Nuts	N	8 ± 11	(0–80)	0.6%	94.4%
21	Egg plant	V	8 ± 7	(0–55)	0.5%	95.0%
22	Fruit juice	F	7 ± 12	(0–102)	0.5%	95.4%
23	Apple/banana	F	7 ± 5	(0–25)	0.5%	95.9%
24	Green soybean	V	6 ± 7	(0–52)	0.4%	96.3%
25	Sesame	N	6 ± 7	(0–54)	0.4%	96.7%
26	Cocoa/chocolate drink	B	5 ± 21	(0–186)	0.3%	97.0%
27	Potato	Po	4 ± 4	(0–29)	0.3%	97.3%
28	Cabbage	V	4 ± 2	(0–15)	0.2%	97.5%
29	Soy milk	P	3 ± 14	(0–162)	0.2%	97.7%
30	*Udon* noodle	C	3 ± 3	(0–21)	0.2%	98.0%
31	Japanese radish/turnip	V	3 ± 3	(0–23)	0.2%	98.2%
32	Prune	F	3 ± 12	(0–72)	0.2%	98.4%
33	Parsley/perilla	V	3 ± 4	(0–31)	0.2%	98.6%
34	Chinese noodle	C	3 ± 3	(0–22)	0.2%	98.7%
35	Curry powder	S	2 ± 2	(0–18)	0.2%	98.9%
36	Ginger	V	2 ± 2	(0–24)	0.2%	99.1%
37	Rice	C	2 ± 1	(0–7)	0.1%	99.2%
38	Pasta	C	2 ± 2	(0–24)	0.1%	99.3%
39	Other teas	B	2 ± 8	(0–54)	0.1%	99.5%
40	Laver	A	2 ± 2	(0–15)	0.1%	99.6%
**Men (*n* = 569)**						
1	Coffee	B	652 ± 520	(0–2613)	43.4%	43.4%
2	Green tea	B	400 ± 354	(0–1380)	26.6%	69.9%
3	Beer	B	46 ± 64	(0–513)	3.0%	73.0%
4	Red wine	B	34 ± 157	(0–2243)	2.3%	75.3%
5	Tomato vegetable juice	V	29 ± 45	(0–311)	1.9%	77.2%
6	Spinach/broccoli	V	26 ± 18	(0–107)	1.7%	78.9%
7	Soy sauce	S	24 ± 7	(10–51)	1.6%	80.4%
8	Buckwheat noodle	C	23 ± 27	(0–162)	1.6%	82.0%
9	*Natto*	P	21 ± 23	(0–142)	1.4%	83.4%
10	Black tea	B	20 ± 56	(0–432)	1.4%	84.7%
11	*Tofu*/fried *tofu*	P	20 ± 13	(0–75)	1.3%	86.1%
12	Oolong tea	B	19 ± 54	(0–468)	1.3%	87.3%
13	Onion/welsh onion	V	17 ± 12	(0–70)	1.1%	88.5%
14	Orange	F	16 ± 19	(0–121)	1.1%	89.6%
15	*Miso*	S	15 ± 10	(0–61)	1.0%	90.5%
16	Bread	C	14 ± 9	(0–46)	1.0%	91.5%
17	Chocolate	Co	14 ± 27	(0–390)	0.9%	92.4%
18	Barley tea	B	13 ± 23	(0–108)	0.9%	93.3%
19	Strawberry	F	11 ± 14	(0–111)	0.7%	94.0%
20	Nuts	N	8 ± 11	(0–80)	0.5%	94.5%
21	Egg plant	V	8 ± 7	(0–55)	0.5%	95.1%
22	Fruit juice	F	7 ± 12	(0–102)	0.5%	95.5%
23	Apple/banana	F	7 ± 5	(0–25)	0.5%	95.9%
24	Green soybean	V	6 ± 7	(0–52)	0.4%	96.4%
25	Sesame	N	5 ± 6	(0–54)	0.4%	96.7%
26	Cabbage	V	4 ± 4	(0–29)	0.3%	97.0%
27	Cocoa/chocolate drink	B	4 ± 19	(0–186)	0.3%	97.3%
28	Potato	Po	4 ± 2	(0–15)	0.2%	97.5%
29	*Udon* noodle	C	3 ± 3	(0–21)	0.2%	97.8%
30	Japanese radish/turnip	V	3 ± 3	(0–23)	0.2%	98.0%
31	Soy milk	P	3 ± 14	(0–162)	0.2%	98.2%
32	Prune	F	3 ± 12	(0–72)	0.2%	98.4%
33	Chinese noodle	C	3 ± 3	(0–22)	0.2%	98.6%
34	Parsley/perilla	V	3 ± 4	(0–31)	0.2%	98.8%
35	Curry powder	S	2 ± 2	(0–18)	0.2%	98.9%
36	Ginger	V	2 ± 2	(0–24)	0.2%	99.1%
37	Rice	C	2 ± 1	(0–7)	0.2%	99.2%
38	Pasta	C	2 ± 2	(0–24)	0.1%	99.4%
39	Laver	A	2 ± 2	(0–15)	0.1%	99.5%
40	Other teas	B	2 ± 7	(0–54)	0.1%	99.6%
**Women (*n* = 41)**						
1	Coffee	B	549 ± 464	(0–2250)	41.4%	41.4%
2	Green tea	B	359 ± 319	(0–1380)	27.1%	68.4%
3	Black tea	B	61 ± 112	(0–432)	4.6%	73.0%
4	Spinach/broccoli	V	25 ± 18	(3–85)	1.9%	75.0%
5	Tomato vegetable juice	V	23 ± 38	(0–104)	1.7%	76.7%
6	*Tofu*/fried *tofu*	P	22 ± 13	(2–77)	1.7%	78.3%
7	Oolong tea	B	21 ± 63	(0–263)	1.6%	79.9%
8	Barley tea	B	21 ± 27	(0–88)	1.6%	81.5%
9	Orange	F	17 ± 16	(0–53)	1.3%	82.7%
10	Soy sauce	S	17 ± 5	(8–28)	1.3%	84.0%
11	*Natto*	P	16 ± 19	(0–62)	1.2%	85.2%
12	Onion/welsh onion	V	14 ± 11	(2–61)	1.1%	86.3%
13	Bread	C	13 ± 7	(0–32)	1.0%	87.3%
14	*Miso*	S	13 ± 11	(0–53)	1.0%	88.3%
15	Chocolate	Co	13 ± 15	(0–61)	1.0%	89.2%
16	Red wine	B	12 ± 29	(0–123)	0.9%	90.2%
17	Strawberry	F	12 ± 13	(1–54)	0.9%	91.0%
18	Egg plant	V	11 ± 12	(0–55)	0.8%	91.8%
19	Buckwheat noodle	C	10 ± 12	(0–50)	0.8%	92.6%
20	Nuts	N	9 ± 11	(0–40)	0.7%	93.3%
21	Fruit juice	F	9 ± 16	(0–51)	0.7%	94.0%
22	Cocoa/chocolate drink	B	9 ± 33	(0–186)	0.7%	94.7%
23	Apple/banana	F	8 ± 5	(1–22)	0.6%	95.3%
24	Sesame	N	8 ± 8	(0–36)	0.6%	95.9%
25	Soy milk	P	6 ± 15	(0–54)	0.5%	96.4%
26	Other teas	B	6 ± 12	(0–36)	0.4%	96.8%
27	Green soybean	V	5 ± 5	(0–17)	0.4%	97.2%
28	Cabbage	V	4 ± 3	(0–10)	0.3%	97.5%
29	Parsley/perilla	V	4 ± 6	(0–31)	0.3%	97.8%
30	Beer	B	3 ± 9	(0–46)	0.3%	98.1%
31	Potato	Po	3 ± 2	(0–11)	0.2%	98.3%
32	Ginger	V	3 ± 2	(0–8)	0.2%	98.5%
33	Japanese radish/turnip	V	2 ± 2	(0–10)	0.2%	98.7%
34	Prune	F	2 ± 11	(0–72)	0.2%	98.8%
35	Curry powder	S	2 ± 1	(0–3)	0.2%	99.0%
36	*Udon* noodle	C	2 ± 2	(0–9)	0.1%	99.1%
37	Laver	A	2 ± 2	(0–5)	0.1%	99.3%
38	Pasta	C	2 ± 2	(0–15)	0.1%	99.4%
39	Rice	C	2 ± 1	(0–4)	0.1%	99.5%
40	Chinese noodle	C	1 ± 1	(0–7)	0.1%	99.6%

B, beverages; V, vegetables; S, seasonings; C, cereals; P, pulses; F, fruits; Co, confectioneries; N, Nuts and seeds; Po, Potatoes; A, Algae.

The top 10 items (composed of four food items and six beverages) and the top 20 items (12 food and eight beverages) accounted for more than 80% and 90% of the cumulative daily consumption of polyphenols, respectively. As for food, although its contribution was small, spinach/broccoli (1.7%), soy sauce (1.6%), buckwheat noodles (1.5%), *natto* (*i.e.*, fermented soybeans) (1.4%), *tofu* (*i.e.*, soybean curd)/fried *tofu* (1.3%), onion/welsh onion (1.1%), *miso* (*i.e.*, fermented soybean paste) (0.97%), bread (0.96%) and chocolate (0.93%) contributed to the polyphenol intake. Soybean products such as soy sauce, *natto*, *tofu*/fried *tofu*, *miso*, and soy milk together accounted for 5.4%, as the third largest contributor after coffee and green tea.

### 3.3. Association between Polyphenol Intake and Micronutrient Intake

The correlation coefficients of polyphenol intake with energy-adjusted micronutrient intakes are shown in Table 4. We calculated the energy-adjusted values of micronutrient intake by the density method. We found that the polyphenol intake was not strongly associated with any of the micronutrients. Highest correlations were found for niacin, magnesium and potassium (*r* = 0.266, 0.245 and 0.223, respectively).

**Table 4:** Correlation coefficients of energy-adjusted micronutrient intakes with polyphenol intake.

Nutrients	*r*
Energy	0.317
Niacin *	0.266
Magnesium *	0.245
Potassium *	0.223
Manganese *	0.187
Folate *	0.182
Vitamin C *	0.180
Iron *	0.156
Riboflavin *	0.150
Copper *	0.126
Fiber *	0.123
β-Carotene equivalent *	0.118
Vitamin K *	0.117
Phosphorus *	0.117
α-Tocopherol *	0.107
Vitamin B6 *	0.106
Vitamin B12 *	0.106
Pantothenic acid *	0.104
Vitamin A (retinol equivalent) *	0.097
Calcium *	0.087
Thiamin *	0.085
Zinc *	0.078
Vitamin D *	0.060
Retinol *	0.050
Sodium *	0.012

Values are the Spearman’s correlation coefficients. * Energy-adjusted micronutrient intakes by the density method.

## 4. Discussion

Our present findings revealed that in a population of elderly Japanese, mostly (93%) men, consumed on average 1492 ± 665 mg/person/day of polyphenol (1503 ± 669 mg/day in the men and 1326 ± 583 mg/day in the women). The dietary polyphenol intake was largely composed of beverages, which accounted for 79% of the intake. Coffee was the largest source of polyphenol intake at 43%, followed by green tea at 27%. This is in line with our previous study showing that beverages accounted for approx. 80% of the total intake, and that coffee and green tea were the major sources of dietary polyphenol [30].

Compared to our earlier study, which demonstrated that middle-aged Japanese women consumed 841 ± 406 mg/day of polyphenol [30], the elderly Japanese in the present study consumed a greater amount of polyphenol. Our recent study using a beverage survey of approx. 10,000 Japanese men and women indicated that the polyphenol intake from beverages was larger in the elderly than in the young or middle-aged and greater among the men than the women, and that the polyphenol intake was influenced by the amounts of coffee and green tea consumed [30]. The elderly women in this study consumed larger amounts of polyphenol compared to the middle-aged women in our previous study. Therefore, polyphenol intake of elderly Japanese might be higher than that of Japanese adult for both men and women.

It is also noteworthy that the daily polyphenol consumption varied considerably among the individuals, ranging from 183 to 4854 mg/day (Figure 1). The polyphenol intake from food showed less individual variation compared to the polyphenol intake from beverages. Coffee and green tea consumption had large impacts on the subjects’ polyphenol intake, and the amount of coffee and green tea consumption varied according to individual preference. Our studies suggest that the amount of beverage consumption, especially coffee and green tea, is a principal factor in the individual variation of polyphenol intake among the Japanese.

As shown in Table 3, the top 10 food and beverage items accounted for >80% of the total polyphenol intake, and the top 20 items accounted for >90%, indicating that a very limited variety of items could contribute to the dietary polyphenol intake by elderly Japanese. Seventeen of the top 20 food and beverage items identified in the present study were in common with those identified in middle-aged women in our previous study (*i.e.*, coffee, green tea, beer, red wine, tomato vegetable juice, soy sauce, black tea, buckwheat noodle, *natto*, *tofu*, oolong tea, onion, orange, *miso*, bread, chocolate, and barley tea), although the ranking order was partly different: the rankings of beer and red wine were higher in the elderly subjects. We found that the daily polyphenol intake in the subjects could be estimated by the assessment of about 10–20 food and beverage items with an accuracy rate at 80%–90%. Our original database containing the polyphenol contents of 67 items covered the major contributors to polyphenol intake in Japan.

Several studies have reported daily polyphenol consumption in European countries, showing that it was 863 ± 415 mg/day in Finnish [38], 1193 ± 510 mg/day in French [10], 820 ± 323 mg/day in Spanish [39], and 1756.5 ± 695.8 mg/day in Polish [40], which have used the Phenol-Explorer database or similar quantitative value measured by HPLC methods. In order to compare our data to those in Europe, we calculated total polyphenol intake in this study’s subjects using the Phenol-Explorer database. The total polyphenol intake was estimated to be 961 ± 452 mg/day, although 19 food and beverage items unlisted in the database could not be included for the calculation. The estimated value was remarkably lower than the value obtained by our database. The discrepancy might be due to the fact that the value for the total poplyphenol in coffee, the largest source of pholyphenols, is lower in Phenol-Explorer (123.6 mg/100 g, as calculated the usage of the two major cultivar Arabica and Robusta coffee beverage at 70% and 30%, respectively, based on Japanese statics of import) than our database (200 mg/100 g). Coffee contains a mixture of coffee polyphenols including 5-caffeoyl quinic acid (5-CQA), other chlorogenic acid groups, and other phenolic compounds generated through roasting process. In the HPLC methods, it is difficult to measure all polyphenol molecules due to the limited standard samples, which causes to underestimate total polyphenol contents. In our unpublished data, 5-CQA and the other simple 7 chlorogenic acids made up 10% and 20% of total polyphenol, respectively, in the soluble coffee sample. On the contrary, the Folin-Ciocalteu method has advantages to roughly estimate overall amounts of polyphenols. Additionally, the lack of some Japanese food items, such as *miso*, buckwheat noodle and sweet bean paste in Phenol-Explorer might be another cause for the discrepancy. On the other hand, total polyphenol contents of some foods such as potato, apple and grape in the Phenol-Explorer database are much higher than that in our database. Japanese typically peel the skin of potato and fruits off and do not consume it. Such eating manner of food and/or difference in harvest or breeding manner should be different among countries and societies. Our original database for polyphenol contents is valuable to properly estimate total dietary polyphenol intakes in Japanese populations. 

In this study, the elderly Japanese subjects consumed polyphenols largely from beverages, and less from vegetables, cereals, and fruits, while they were the major contributors to polyphenol intake in the other European countries. For example, French adults consumed a certain amount of polyphenols from fruits (17%), vegetables (7%) and cereals (4%). On the other hand, soybeans play an important part in the polyphenol intake in the Japanese diet. It was reported that soy consumptions and isoflavone intakes were high in the Japanese population compared to those of other countries [41,42]. Food items made from soybeans such as *tofu* and *natto* and seasonings made from soybeans (*i.e.*, soy sauce and *miso*) accounted for 5.4% of the total polyphenol intake in the present study.

As mentioned above, accumulating evidence indicates that polyphenol intake may contribute to reducing the risk of the development of several diseases and their related mortality rates [14,15,16,17,18,19,20,21,22,23,24,25,26]. However, it is still unclear whether these benefits for human health are due to the intakes of polyphenols and/or to the accompanying nutrients. In the present study, we also evaluated the relationship between polyphenol intake and nutritional intake, and interestingly, we found that the subjects’ polyphenol intake was not associated strongly with the intake of any micronutrients, but it was very weakly associated with the intake of some micronutrients (*i.e.*, niacin, magnesium and potassium). These findings may have been obtained because the Japanese are largely dependent on coffee and green tea for polyphenol consumption. In fact, weak but statistically significant associations between total polyphenol intake and some micronutrients such as magnesium, vitamin B6 and vitamin K were detected when these two beverages were excluded from the analysis (*r* = 0.291, 0.289 and 0.280, respectively). In countries such as Spain and France, the consumption of dietary polyphenols could be accompanied by that of specific nutrients derived from the consumption of fruits and/or vegetables. In future epidemiological studies, it will be necessary to determine the links between dietary polyphenol and nutrients in order to clarify the health benefits of a polyphenol-rich diet.

Some limitations of our study should be mentioned. First, the subjects in this study were all recruited from among the retired employees of a food manufacturing company, which may have caused a bias in the subject population who drank greater amounts of coffee (approx. +100 mL/day) compared to the same age-group we observed in our previous study [31]. Although this study was not population-based and showed slightly higher coffee consumption, the observation we found in our series of studies showing that coffee and green tea are the largest sources of polyphenols in the Japanese diet remains consistent. Moreover, we used the original FFQ and polyphenol content database for estimating dietary polyphenol intake. In our unpublished data, total polyphenol intake estimated by the FFQs was significantly correlated with those obtained by the dietary records. Our database consists of a limited number of foods, but its weight-based coverage of the consumption of vegetable and fruit items with polyphenol content information was 94%–95% [30]. Finally, the number of women in the present study was very small at 6.7%.

## 5. Conclusions

In conclusion, the present study showed that a population of elderly Japanese (mostly men) consumed 1492 ± 665 mg/day of polyphenols on average, which is slightly higher than previous data in Japanese adults, and coffee and green tea were the largest sources of polyphenols in their daily life. Our data will be valuable as additional baseline data of polyphenol intake in the Japanese and for future research focused on the relationship between polyphenols and their health benefits for humans.
